# Dabrafenib; Preclinical Characterization, Increased Efficacy when Combined with Trametinib, while BRAF/MEK Tool Combination Reduced Skin Lesions

**DOI:** 10.1371/journal.pone.0067583

**Published:** 2013-07-03

**Authors:** Alastair J. King, Marc R. Arnone, Maureen R. Bleam, Katherine G. Moss, Jingsong Yang, Kelly E. Fedorowicz, Kimberly N. Smitheman, Joseph A. Erhardt, Angela Hughes-Earle, Laurie S. Kane-Carson, Robert H. Sinnamon, Hongwei Qi, Tara R. Rheault, David E. Uehling, Sylvie G. Laquerre

**Affiliations:** 1 GlaxoSmithKline Department of Oncology Biology, Collegeville, Pennsylvania, United States of America; 2 GlaxoSmithKline Department of Enzymology & Mechanistic Pharmacology, Collegeville, Pennsylvania, United States of America; 3 GlaxoSmithKline Portfolio Management, Collegeville, Pennsylvania, United States of America; 4 GlaxoSmithKline Department of Safety Assessment, King of Prussia, Pennsylvania, United States of America; 5 GlaxoSmithKline Department of Screening & Compound Profiling, GlaxoSmithKline, Durham, North Carolina, United States of America; 6 GlaxoSmithKline Department of Biological Reagents & Assay Development, King of Prussia, Pennsylvania, United States of America; 7 GlaxoSmithKline Department of Medicinal Chemistry, GlaxoSmithKline, Durham, North Carolina, United States of America; The Moffitt Cancer Center & Research Institute, United States of America

## Abstract

Mitogen-Activated Protein Kinase (MAPK) pathway activation has been implicated in many types of human cancer. BRAF mutations that constitutively activate MAPK signalling and bypass the need for upstream stimuli occur with high prevalence in melanoma, colorectal carcinoma, ovarian cancer, papillary thyroid carcinoma, and cholangiocarcinoma. In this report we characterize the novel, potent, and selective BRAF inhibitor, dabrafenib (GSK2118436). Cellular inhibition of BRAF^V600E^ kinase activity by dabrafenib resulted in decreased MEK and ERK phosphorylation and inhibition of cell proliferation through an initial G_1_ cell cycle arrest, followed by cell death. In a BRAF^V600E^-containing xenograft model of human melanoma, orally administered dabrafenib inhibited ERK activation, downregulated Ki67, and upregulated p27, leading to tumor growth inhibition. However, as reported for other BRAF inhibitors, dabrafenib also induced MAPK pathway activation in wild-type BRAF cells through CRAF (RAF1) signalling, potentially explaining the squamous cell carcinomas and keratoacanthomas arising in patients treated with BRAF inhibitors. In addressing this issue, we showed that concomitant administration of BRAF and MEK inhibitors abrogated paradoxical BRAF inhibitor-induced MAPK signalling in cells, reduced the occurrence of skin lesions in rats, and enhanced the inhibition of human tumor xenograft growth in mouse models. Taken together, our findings offer preclinical proof of concept for dabrafenib as a specific and highly efficacious BRAF inhibitor and provide evidence for its potential clinical benefits when used in combination with a MEK inhibitor.

## Introduction

The MAPK signal transduction pathway plays a central role in cellular growth, differentiation, and stress response [1]–[5]. This pathway is normally activated by the binding of extracellular growth factors to membrane-bound receptors, which then recruit intracellular proteins to the cell membrane, leading to the activation of the small guanosine triphosphate-binding protein, RAS. Consequently, RAS adopts an activated conformation that stimulates downstream signalling, resulting in the phosphorylation and activation of ERK, which regulates a wide range of cellular processes. However, this pathway can be constitutively activated by mutation of specific proteins, including BRAF. Such activating mutations appear to mimic regulatory phosphorylation of BRAF and increase its kinase activity compared with the wild-type protein [6]. Over 45 cancer-associated BRAF mutations have been identified [7] with a high frequency in specific cancers, including 40–60% of melanoma [6], 30–50% of papillary thyroid, 5–20% of colorectal, and ∼30% of ovarian cancer [7]. Approximately 90% of all BRAF mutations identified in human cancers are a T1799A transversion in exon 15, which results in a V600E amino acid substitution and BRAF kinase activation [7], [8]. The high frequency of activating mutations in tumors and ensuing MAPK pathway addiction make BRAF an attractive therapeutic target, where inhibition of the kinase activity of BRAF^V600E^ and other activated BRAF mutants could provide an effective therapy.

BRAF inhibitors with diverse levels of selectivity have been identified and clinically tested [9]–[13]. Despite demonstrating therapeutic activity, a clinical increase in squamous cell carcinoma (SCC) incidence has been associated with treatment using sorafenib [14]–[17], PLX4032 [18], and GSK2118436 (dabrafenib) [11]. Increased ERK phosphorylation in wild-type cells exposed to these inhibitors, caused by feedback upregulation of the MAPK pathway, was proposed to be responsible for increased cell proliferation that may lead to SCC growth [19]–[22].

We have identified a BRAF inhibitor, dabrafenib [23], and here characterize its preclinical activity with high potency, selectivity, and inhibition of human tumor xenograft growth. We also demonstrate that increased phospho-ERK in wild-type BRAF cells, as a result of exposure to dabrafenib, is CRAF-dependent and can be abrogated by MEK inhibition. Furthermore, we demonstrate that co-administration of BRAF and MEK inhibitors improves both the safety, by reducing the occurrence of skin lesions, and the *in vivo* activity profile, by reducing tumor regrowth, over that of a BRAF inhibitor alone in rodents.

## Materials and Methods

### Proteins

Various BRAF orthologs (human, cynomolgus monkey, dog, rat) were cloned in-house. Human BRAF^V600E^ was cloned from the A375P cell line. All BRAF orthologs were sub-cloned into the Gateway® vector system, and wild-type human BRAF (residues 1–766) subsequently underwent site-directed mutagenesis to generate the V600D and V600K mutants. Full-length BRAF genes were tagged with 6×His-SBP and transiently transfected in a HEK293F expression system (Invitrogen K9000-01 protocol). Transfected cells were incubated at 37°C, 5% CO_2_ on a shaker at 80 r.p.m. for 48 h and harvested by centrifugation. BRAF proteins were purified by UltraLink®-immobilized Streptavidin affinity and Superdex™ 200 size exclusion chromatography.

Baculovirus-expressed GST-tagged CRAF truncate (residues 306–648), containing Y340D/Y341D mutations for constitutive kinase activity, was obtained from Upstate/Millipore.

N-terminal GST-tagged MEK1 was expressed in BL21[DE3]/pRR692 cells and purified by Glutathione Sepharose™ 4FF and Q-Sepharose™ chromatography. The GST tag was cleaved with TEV protease and MEK1 was purified on a Superdex™ 200 column.

### Enzymology

The activity of dabrafenib against RAF kinases was evaluated using BRAF/CRAF-activated MEK ATPase coupled assays [24]. Inhibitor mode of action was demonstrated in a fluorescence polarization competition binding assay using an in-house TAMRA-labelled, ATP-competitive small molecule ligand, the K_d_ of which was determined as 0.9 nM and 3.6 nM for human wild-type and BRAF^V600E^, respectively. Incubations were carried out over a range of dabrafenib concentrations for 120 min at room temperature in 50 mM HEPES-NaOH pH 7.3, 1 mM CHAPS, 10 mM MgCl_2_, and 1 mM DTT, containing 1 nM ligand and 2 nM wild-type or 4 nM V600E BRAF, after which fluorescence anisotropy was measured using a PerkinElmer EnVision® reader.

### Cell Lines

All cell lines were obtained from the American Type Culture Collection (ATCC) except YUMAC cells, which were purchased from the Yale Dermatology Cell Culture Facility. All cells were grown at 37°C, 5% CO_2_ in the recommended media, supplemented with 10% FBS (SAFC Biosciences) and 1% penicillin/streptomycin (Gibco). BRAF and RAS mutation status were verified for all cell lines by in-house sequencing.

### Antibodies

Antibodies towards the indicated proteins were purchased from the following sources: ERK1/2, pERK (pY204), MEK1/2, pMEK (pS218, pS222) (Santa Cruz Biotechnology), ppERK (pT202, pY204) (Cell Signaling Technology & mouse clone MAPK-YT from Sigma-Aldrich), Ki67 (mouse clone MIB-1), p27^Kip1^ (mouse clone Kip-1) (DakoCytomation), goat anti-rabbit, goat anti-mouse, donkey anti-rabbit, donkey anti-goat secondaries (LI-COR Biosciences), donkey anti-goat (Invitrogen), and sheep anti-rabbit (Rockland). Mouse control IgG for immunohistochemistry was from DakoCytomation.

### Western Blot Analysis

Cells were seeded in 6-well plates at 150 000 to 300 000 per well, grown overnight at 37°C, 5% CO_2_, and treated with 0.2% DMSO or dabrafenib in 0.2% DMSO for the indicated time, or 1 h if not specified. Cells were lysed in 25 mM Tris-HCl pH 7.5, 2 mM EDTA, 2 mM EGTA, 1% Triton X-100, 0.1% SDS, 50 mM sodium-β-glycerophosphate, 2 mM Na_3_VO_4_, 12.5 mM sodium pyrophosphate, 2× Complete EDTA-free mini protease inhibitor tablets (Roche), 2× phosphatase inhibitor cocktails I and II (Sigma-Aldrich). Protein concentrations were determined by Bio-Rad DC™ or dye-based protein assays and lysates were resolved by SDS-PAGE (Invitrogen NuPAGE®) and transferred to PVDF membranes. Membranes were blocked with Odyssey® (LI-COR Biosciences) blocking buffer and incubated with primary antibodies overnight at 4°C. Blots were washed with PBS containing 0.1% Tween-20, incubated with secondary antibodies, and washed again prior to signal detection on a LI-COR Odyssey® reader.

### In-cell Western Analysis

Cells were seeded in 96-well plates at 20 000 to 30 000 per well, grown overnight at 37°C, 5% CO_2_, and treated with 0.2% D DDMSO or dabrafenib in 0.2% DMSO for 1 h, fixed with 3.7% formalin in PBS, permeabilized with 0.1% Triton X-100 in PBS, blocked with Odyssey® (LI-COR Biosciences) blocking buffer, and probed for ERK and pERK. They were then washed with PBS containing 0.1% Tween-20, incubated with secondary antibodies, washed again, and fluorescence was detected on a LI-COR Aerius™ reader.

### siRNA Studies

siRNA complexes were prepared per the manufacturer's instructions using Dharmacon RNAi duplex (Thermo Fisher Scientific), Lipofectamine™ RNAiMAX™ (Invitrogen), and Opti-MEM® (Gibco) and allowed to form at room temperature for at least 40 min. Volumes of 1 mL of RNAi complex were added to each well of a plate with 150 000 cells in complete medium (no antibiotic) and incubated at 37°C, 5% CO_2_ for the indicated times. Data shown are representative of two different studies using pooled siRNA and a third study using a shRNA against a different sequence for each targeted transcript.

### Cell Growth Assay

Cells were seeded at 500 to 2 000 per well in black 384-well plates (Greiner), incubated overnight at 37°C, 5% CO_2_, and treated with 0.2% D DDMSO or dabrafenib in 0.2% DMSO for 72 h. Growth was measured with CellTiter-Glo® (Promega) reagent per the manufacturer’s protocol, using a PerkinElmer EnVision™ reader at t = 0 and 72 h. IC_50_ values for cell growth inhibition (gIC_50_) were determined.

### Cell Cycle Analysis

Cells were seeded in 96-well plates at 3 000 per well, incubated overnight at 37°C, 5% CO_2_, and treated with 0.2% D MSO or dabrafenib in 0.2% DMSO for 24, 48, or 72 h. Cells were permeabilized, DNA was stained with propidium iodide [25], and samples were read on a BD LSRII flow cytometer. Data were analyzed using the cell cycle platform of FlowJo v6.1.1 software.

### Caspase Activation

Cells were seeded in white 96-well plates (NUNC) at 2 500 per well, incubated overnight at 37°C, 5% CO_2_, and treated with 0.2% D MSO or dabrafenib in 0.2% DMSO. At selected times, caspase-3/7 activity and cell density were determined using Caspase-Glo® (Promega) and CellTiter-Glo® reagents, respectively, using a PerkinElmer EnVision™ reader. Caspase activity was normalized to cell density and percent induction over the DMSO control was determined.

### Tumor Xenograft Studies

Cells were implanted subcutaneously in female CD1 *nu/nu* mice and grown to form tumors. When tumors reached 150 mm^3^ to 200 mm^3^, animals were treated with dabrafenib, trametinib (GSK1120212), both agents, or vehicle alone by daily oral gavage of 0.5% hydroxypropylmethylcellulose (Sigma-Aldrich), 0.2% Tween 80 in pH 8.0 distilled water at 0.2 mL per 20 g of body weight for the duration stipulated. Tumor volumes were estimated twice weekly from two-dimensional caliper measurements using the prolate ellipsoid equation 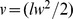, where *v* = volume (mm^3^), *l* = length (mm), and *w* = width (mm), and reported as means for *n* of 7 or 8 mice per group. Tumor growth inhibition represents the percentage volume differential between treated and control tumors at the time when vehicle-treated tumors exceeded a volume of 1 000 mm^3^. Partial regression is defined as a 50% decrease from an individual tumor's starting volume for at least 1 week (three consecutive measurements). Complete regression is defined as a >93% decrease in individual tumor volume for at least 1 week. All animal procedures were conducted in an Association for Assessment and Accreditation of Laboratory Animal Care (AAALAC)-accredited facility at GlaxoSmithKline in accordance with the GlaxoSmithKline Policy on the Care, Welfare, and Treatment of Laboratory Animals and were reviewed and approved by the Institutional Animal Care and Use Committee (IACUC) at GlaxoSmithKline. All efforts were made to minimize suffering and mice were euthanized by carbon dioxide asphyxiation if at least one death occurred per treatment group, if an animal appeared moribund or in distress, or if the tumor became ulcerated.

### Pharmacokinetic Analysis

Compound concentrations were determined from flash-frozen hemolyzed blood samples (blood:water ratio of 1∶1) or tumor homogenates (tumor:water ratio of 1∶4) by HPLC/MS-MS analysis.

### Pharmacodynamic Measurement of Phospho-ERK

Tumors were harvested, homogenized (BD Bioscience Medimachine) in 1 mL each of ice-cold Western Blot Analysis buffer containing 4 mM sodium pyrophosphate, then centrifuged (18 000×g, 15 min, 4°C), and flash-frozen. Samples were analyzed by duplex ELISA (MesoScale Discovery) for total and phospho ERK1/2 per the manufacturer’s instructions using an MSD SI6000 reader.

### Immunohistochemistry

A375P tumors were harvested at 6 h after the sixth daily dose with compound or vehicle, cut into samples of ∼100 mm^3^ in volume, fixed in 10% neutral-buffered formalin (NBF, VWR) for 24 h, and transferred into 70% ethanol prior to paraffin embedding. Paraffin-embedded blocks were sectioned (4 µm to 5 µm) and immunostained for ppERK, Ki67, and p27^Kip1^ by Mosaic Laboratories (Lake Forest, CA).

### Investigative Rat Study to Address BRAF Inhibitor-Induced Skin Lesion Formation by Co-administration of BRAF and MEK Tool Inhibitors

Following acclimation, 24 male Crl:(CD)SD rats (∼9 weeks of age, Charles River Laboratories) were randomized by body weight into four groups of *n* = 6 per group. Animals were dosed for 12 consecutive days by oral gavage with vehicle or 150 mg/kg/d GSK2366297 (a tool BRAF inhibitor) in combination with 0, 0.75, or 1.5 mg/kg/d GSK2091976 (a tool MEK inhibitor) at dose volumes of 10 mL/kg/d (GSK2366297) or 5 mL/kg/d (GSK2091976). Blood samples were collected for toxicokinetic evaluation on day 12 (∼2 h and ∼14 h post-last dose). All efforts were made to minimize suffering. At ∼24 h post-final dose, rats were euthanized by isoflurane anesthesia/exsanguination and necropsied. Skin (left forepaw and hindpaw) was collected into 10% NBF, trimmed, decalcified in Immunocal™ decalcifier until judged complete by palpation, and processed. Stomachs from all study animals and tissues with macroscopic observations were also collected into 10% NBF. All samples were embedded in paraffin wax, sectioned (5 µm), and stained with hematoxylin/eosin for microscopic examination. All animal procedures were conducted in an Association for Assessment and Accreditation of Laboratory Animal Care (AAALAC)-accredited facility at GlaxoSmithKline in accordance with the GlaxoSmithKline Policy on the Care, Welfare, and Treatment of Laboratory Animals and were reviewed and approved by the Institutional Animal Care and Use Committee (IACUC) at GlaxoSmithKline.

## Results

### Dabrafenib is a Potent and Selective BRAF Inhibitor

**Figure 1:**
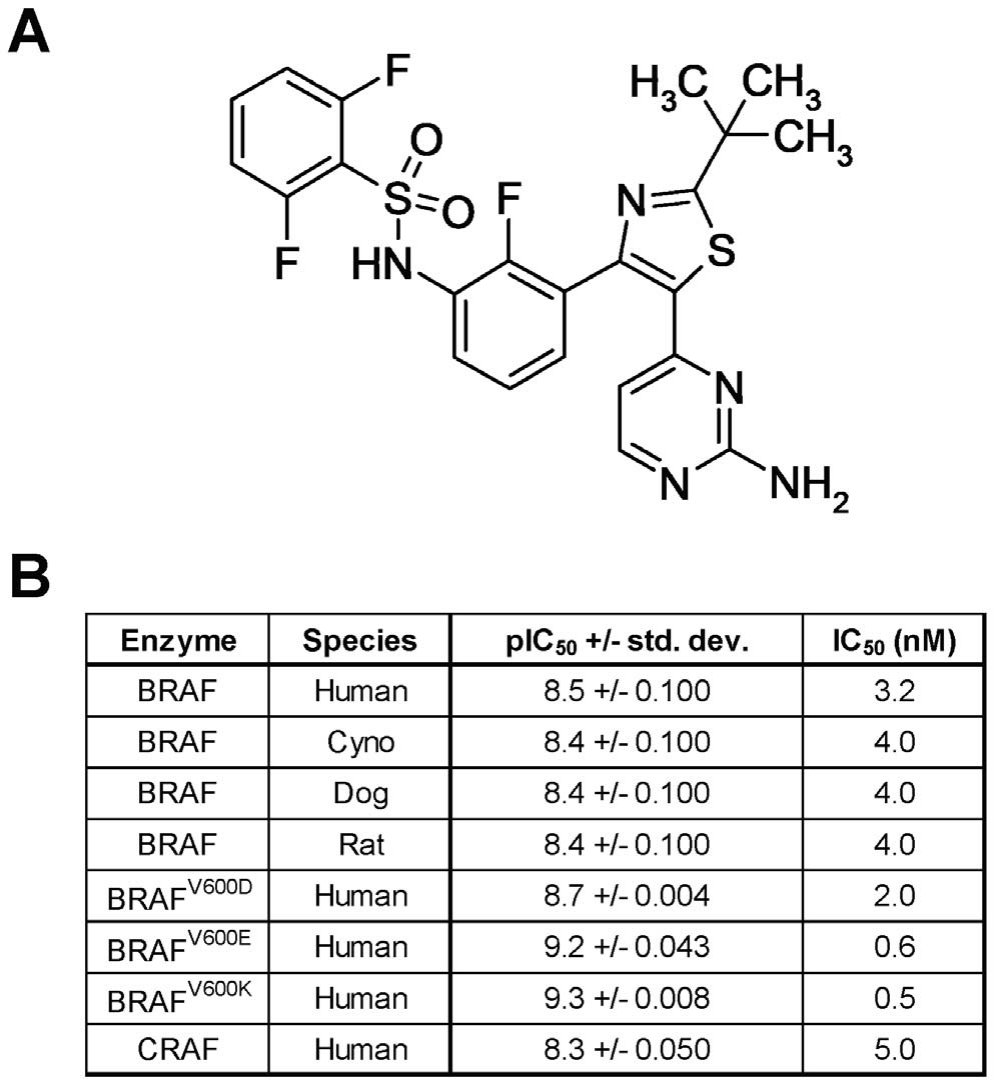
Dabrafenib structure and activity against RAF kinases. Dabrafenib chemical structure (A). Dabrafenib potency (IC_50_) was measured against the kinase activity of various BRAF orthologs, human BRAF mutants, and human CRAF (B).

Dabrafenib (Figure 1A) was identified through a structure-activity-relationship effort described separately [23]. In the current study, dabrafenib displayed similar inhibition of human BRAF, CRAF, and several BRAF orthologs, with slightly higher activity against human BRAF^V600E/K^ (Figure 1B). Binding to BRAF^V600E^ and wild-type BRAF was shown to be ATP-competitive by virtue of its ability to displace a known ATP-competitive ligand (Figure S1). When tested against a panel of 270 kinases, dabrafenib demonstrated selectivity towards RAF kinases, with only 6 additional kinases having an IC_50_<100 nM (Table S1). In cell lines encoding BRAF^V600E^, dabrafenib inhibited pERK and pMEK in a concentration-dependent manner with IC_50_ values of 3 nM and 6 nM, respectively, while total ERK and MEK levels were unchanged (Figure S2).

**Figure 2:**
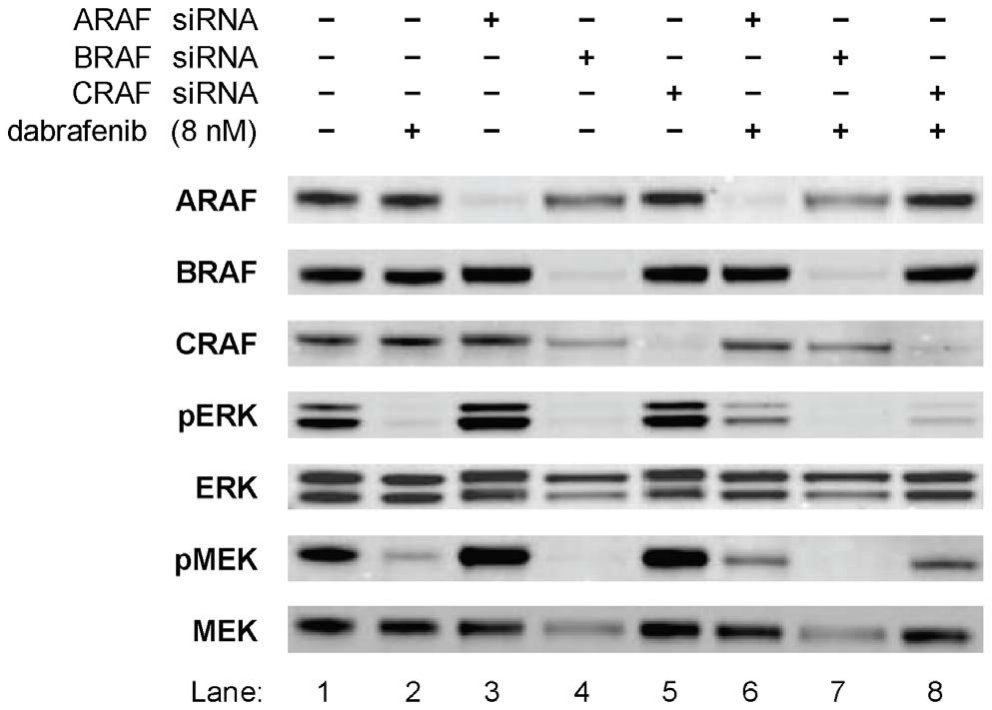
Dabrafenib inhibits MAPK signalling in BRAF^V600E^ cells and is abrogated by ARAF or CRAF depletion. The inhibition of MAPK signalling by dabrafenib in a BRAF^V600E^ cell line was examined in comparison with knockdown of RAF paralogs using siRNA. A375P cells were transfected with the indicated siRNA for 72 h and treated with 8 nM dabrafenib (+) or DMSO control (−) for 1 h. Lysates were immunoblotted for the proteins indicated.

In order to dissect the cellular mechanism of dabrafenib, siRNA studies targeting ARAF, BRAF, or CRAF, alone or in the presence of dabrafenib, were performed in the BRAF^V600E^ cell line, A375P (Figure 2). A dabrafenib concentration of 8 nM, selected to allow detection of subtle inhibitory effects from pathway protein level changes, inhibited pERK and pMEK by >90% (lane 2) while ARAF, BRAF, CRAF, ERK, and MEK levels remained unchanged. By way of comparison, ARAF (lane 3) and CRAF (lane 5) siRNA did not alter pERK or pMEK, while BRAF siRNA (lane 4) inhibited pERK and pMEK, as expected and as demonstrated elsewhere [26], [27], confirming that MEK and ERK activation is exclusively BRAF^V600E^-dependent in a BRAF^V600E^ cell line. Moreover, and in answer to the question posed by the current study, inhibition of MEK and ERK activation by dabrafenib (lane 2) was comparable with that of BRAF siRNA, further demonstrating this compound's ability to inhibit BRAF^V600E^ signalling in cells. Interestingly, ARAF (lane 6) or CRAF (lane 8) knockdown lowered sensitivity to dabrafenib, as judged by increased pERK and pMEK when compared with compound alone (lane 2). Taken together, these data demonstrate that in BRAF^V600E^ cells, MAPK activation is solely driven by BRAF^V600E^ and can be effectively inhibited by dabrafenib.

### Dabrafenib Inhibits Mutant BRAF Cell Line and Tumor Xenograft Growth

**Figure 3:**
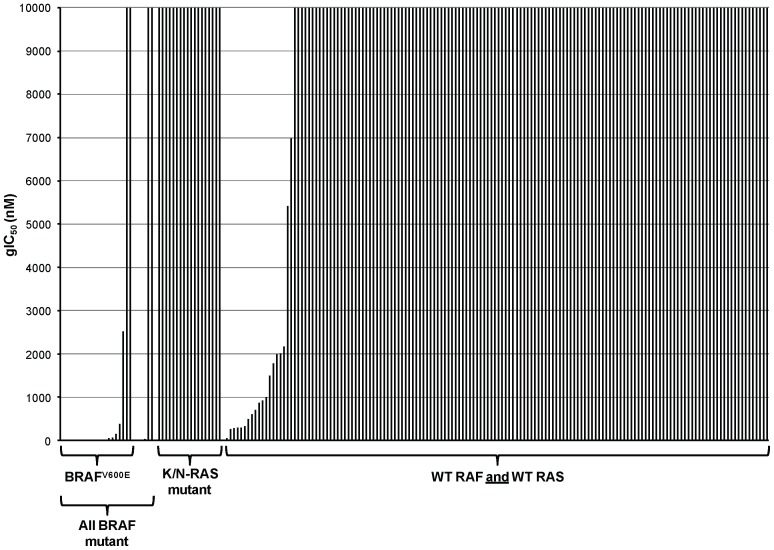
Dabrafenib shows functional selectivity for BRAF^V600D/E/K^ tumor cell growth inhibition. The functional selectivity of dabrafenib was evaluated by measuring the growth of a range of human tumor cell lines of different genetic mutation status. Cells were treated with a concentration range of dabrafenib in a 3-day proliferation assay and growth IC_50_ (gIC_50_) values were measured.

Dabrafenib was tested for its ability to inhibit the growth of 195 tumor cell lines in a 3-day proliferation assay (Figure 3 and Table S2). Sixteen (80%) of the 20 cell lines encoding BRAF^V600E^ were sensitive to dabrafenib (gIC_50_<200 nM). Furthermore, three of the other 5 mutant BRAF cell lines were sensitive to dabrafenib (gIC_50_<30 nM), including WM-115 (BRAF^V600D^) and YUMAC (BRAF^V600K^). However, 133 (88%) of the 152 RAS/RAF wild-type and all 18 mutant RAS cell lines were insensitive to dabrafenib (gIC_50_>10 µM), demonstrating high selectivity for inhibition of the growth of activated mutant BRAF cell lines.

The effect of dabrafenib on cell cycle and induction of apoptosis was analyzed in cell lines encoding BRAF^V600E^ (A375P, SK-MEL-28) or wild-type BRAF (HFF). Dabrafenib arrested the BRAF^V600E^ cells at the G_1_ (2n DNA) phase of the cell cycle, with increased DNA fragmentation (<2n DNA) at higher concentrations, whereas wild-type BRAF cells were unaffected at dabrafenib concentrations up to 10 µM (Figure S3A). While the level of apoptosis can vary between cell lines [28], dabrafenib consistently induced caspase-3/7 activation and apoptosis in these BRAF^V600E^ cells (Figure S3B).

**Figure 4:**
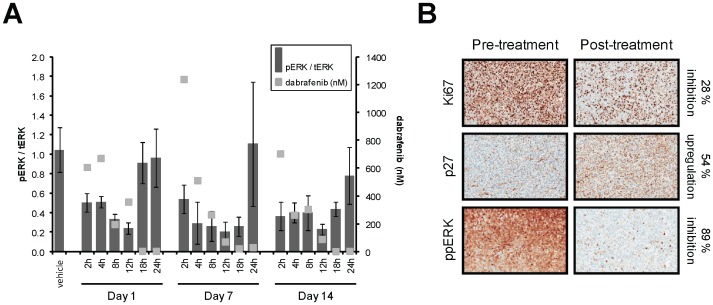
Modulation of pharmacodynamic markers by dabrafenib in BRAF^V600E^ tumors. Mice bearing A375P tumor xenografts were treated orally with 30 mg/kg dabrafenib, once daily for 14 days. Blood and tumors from vehicle- and dabrafenib-treated animals were analyzed for compound concentration and pERK inhibition, respectively (A). Phospho-ERK (pERK) is normalized to total ERK (tERK). Tumors harvested 6h post-last 6^th^ dose were stained for Ki67, p27, and ppERK by immunohistochemistry and compared with pre-treatment controls (B). Data are representative of n = 3 studies and percent changes were calculated from the ratio of positively stained cells following drug treatment to those following vehicle control treatment, each as a percentage of the total cell population.

**Figure 5:**
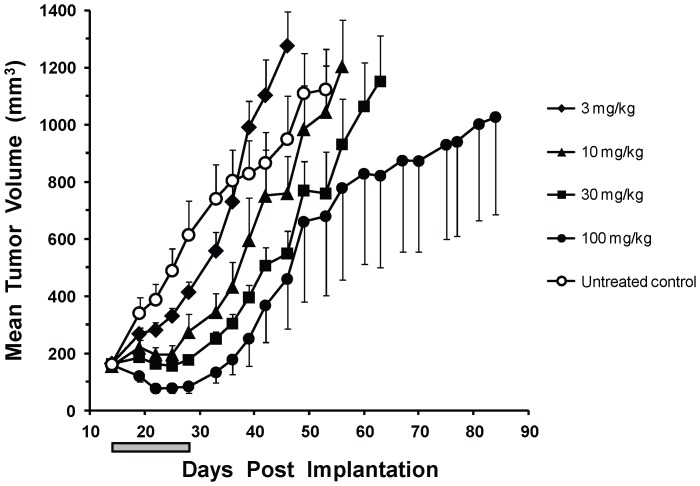
Inhibition of BRAF^V600E^ tumor xenograft growth by dabrafenib. Growth of Colo 205 tumor xenografts was measured in mice during and for a period following oral q.d. × 14 treatment with 0, 3, 10, 30, and 100 mg/kg dabrafenib. Mean tumor volumes are plotted with their standard error mean and 4 partial regressions out of 8 mice were observed at the 100 mg/kg dose after the 14-day treatment period. The 14-day period of dosing is indicated by the shaded gray bar.

The *in vivo* activity of dabrafenib was tested in a BRAF^V600E^ (A375P) human tumor xenograft model in mice. Mice were dosed orally once daily at 30 mg/kg for 14 days and tumors were measured both during and following cessation of treatment. Tumors and blood were harvested for pERK inhibition and dabrafenib concentration measurement, respectively, at days 1, 7, and 14. Dabrafenib is orally bioavailable, doesn’t significantly accumulate after multiple dosing, and causes a reduction of pERK that is sustained for up to 18 h post-dosing after 7 and 14 days of dosing (Figure 4A). Tumors from 6 h post-final dose were stained by immunohistochemistry (Figure 4B) for markers of cell proliferation (Ki67), growth inhibition (p27), and MAPK signalling (pERK). Results support an on-target mechanism for cell growth inhibition by dabrafenib, which elicited a downregulation of pERK and Ki67 by 89% and 28%, respectively, as well as upregulation of p27 by 54%. These changes in tumor growth marker levels translated into efficacy, where dabrafenib showed dose-dependent inhibition of tumor growth in a BRAF^V600E^ (Colo 205) human tumor xenograft model (Figure 5) after a 14-day treatment, with 4 of 8 mice showing partial regressions at 100 mg/kg. Inhibition of tumor growth was significant when compared with the vehicle-treated control at the end of the 14-day dosing period, with p-values of 0.007, 0.00009, and 0.000005 for dabrafenib doses of 10, 30, and 100 mg/kg, respectively. Use of the Colo 205 model demonstrated activity in yet another BRAF^V600E^ cell/tumor type (colorectal cancer), while similar results were also seen in an A375P tumor xenograft model [23] with 4 complete and 2 partial regressions at 100 mg/kg, along with 1 complete and 2 partial regressions at 10 mg/kg from groups of 8 mice. In both cases, drug treatment cessation after 14 days resulted in tumor outgrowth, suggesting a need for chronic therapy for maximum clinical benefit.

### Dabrafenib Induces MAPK Activation in Wild-type RAF Cells

**Figure 6:**
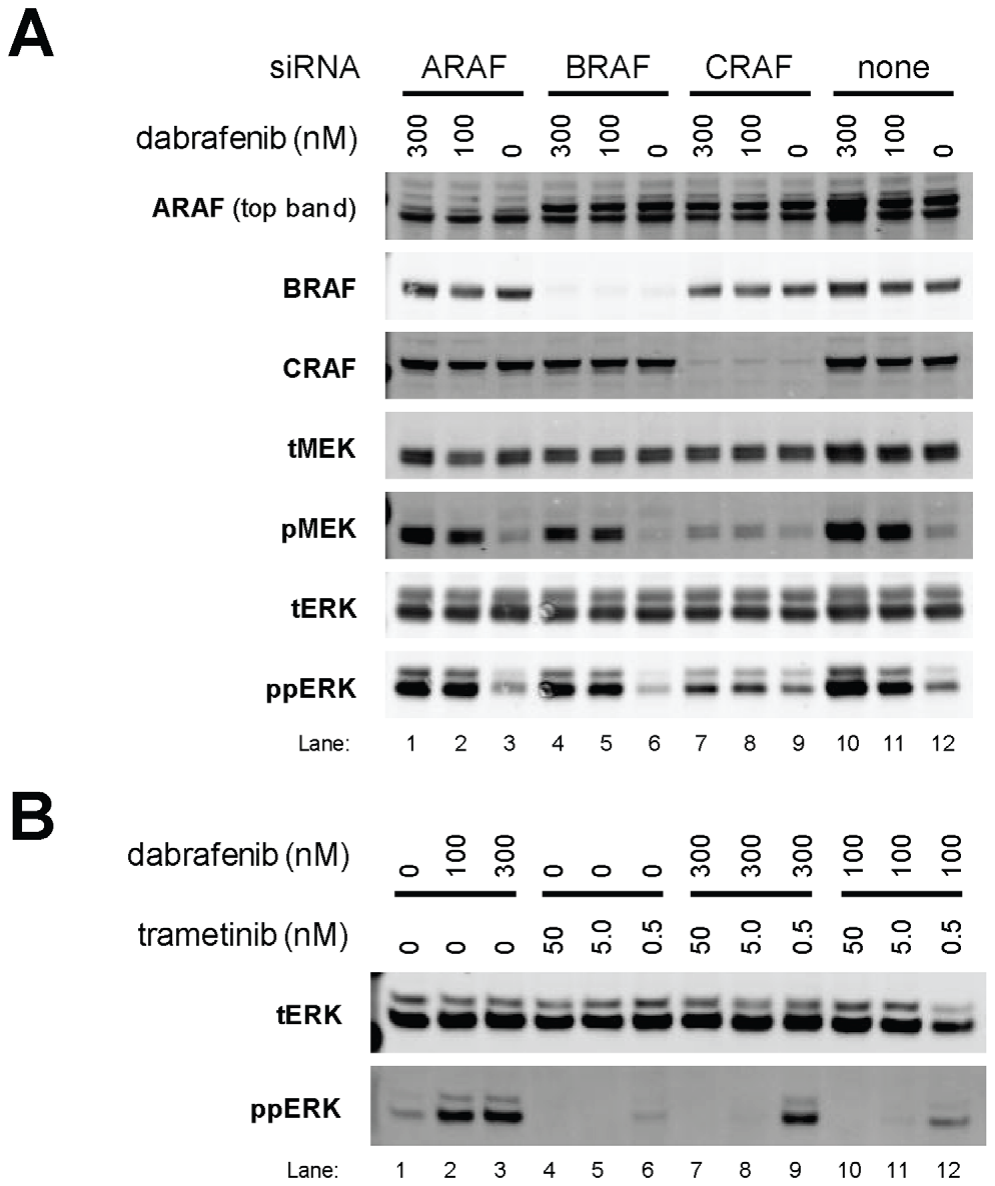
Dabrafenib-induced MAPK activation in wild-type BRAF/mutant RAS cells is CRAF-dependent and abrogated by MEK inhibition. In order to assess the individual contributions of ARAF, BRAF, and CRAF to paradoxical MAPK activation caused by dabrafenib in HCT-116 (wild-type BRAF, mutant KRAS) cells, phospho and total MEK (pMEK, tMEK) and ERK (pERK, tERK) were evaluated by immunoblot after a 72-hour incubation with siRNA towards ARAF, BRAF, or CRAF, or medium (none), and treatment with 0, 100, or 300 nM dabrafenib for 1 h (A). Sensitivity of this paradoxical activation to MEK inhibition was evaluated in HCT-116 cells, following treatment with 0, 100, or 300 nM dabrafenib for 1 h in the presence of 0, 0.5, 5.0, or 50 nM MEK inhibitor (trametinib). Lysates were immunoblotted for total ERK (tERK) and dual-phosphorylated ERK (ppERK) (B).

While dabrafenib can inhibit proliferation via a G_1_ cell cycle arrest, induce apoptosis, and cause tumor regression in BRAF^V600E^ cells, wild-type BRAF tumor cell lines were not responsive to dabrafenib, despite MEK inhibitor sensitivity [29]. Indeed, HCT-116 cells, which encode mutant KRAS and wild-type RAF proteins, are insensitive to dabrafenib (Figure 3 and Table S2) but sensitive to a MEK1/2 inhibitor (GSK1120212, trametinib) with a gIC_50_ of 21 nM [29]. Additionally, HCT-116 cells showed increased pMEK and pERK following dabrafenib treatment at 100 nM or 300 nM (Figure 6A, lanes 11 and 10, respectively) when compared with the DMSO control (lane 12). In order to demonstrate through which RAF protein dabrafenib elicits this elevated MAPK signalling, we depleted individual RAF proteins using siRNA (Figure 6A). Following dabrafenib treatment, MAPK upregulation was not sensitive to ARAF or BRAF knockdown (lanes 1/2 and 4/5, respectively), but was reduced to baseline upon CRAF depletion (lanes 7/8). Consequently, these data suggest that paradoxical MAPK pathway activation by dabrafenib in wild-type RAF and mutant RAS cell lines is CRAF-dependent and that dabrafenib is only active against activated BRAF in cells.

### BRAF/MEK Tool Inhibitor Combination Decreases the Occurrence of Skin Lesions and Dabrafenib/Trametinib Combination Enhances Tumor Growth Inhibition in Rodent Models

It has been proposed that hyperproliferative skin lesions (keratoacanthomas) and in some cases SCC in BRAF inhibitor-treated patients were caused by pERK upregulation in wild-type BRAF cells [20]–[22], with a high RAS mutation frequency [30]. This upregulation is CRAF-dependent, so we hypothesized that combined BRAF/CRAF inhibition might reduce the incidence of hyperproliferative skin lesions. Since no selective, efficacious CRAF inhibitor is available, we tested this hypothesis by combining BRAF (dabrafenib) and MEK (trametinib) inhibitors. First, we demonstrated that dabrafenib-induced pERK upregulation in HCT-116 cells could be abrogated by simultaneous treatment with both inhibitors (Figure 6B). Indeed, pERK upregulation following treatment with 100 or 300 nM dabrafenib alone (lanes 2/3) was abolished by co-treatment with 50 nM or 5 nM trametinib (lanes 7/10 and 8/11, respectively). This concurs with data in Figure 6A, showing that dabrafenib-induced pERK upregulation in HCT-116 cells is MAPK pathway-dependent.

**Figure 7:**
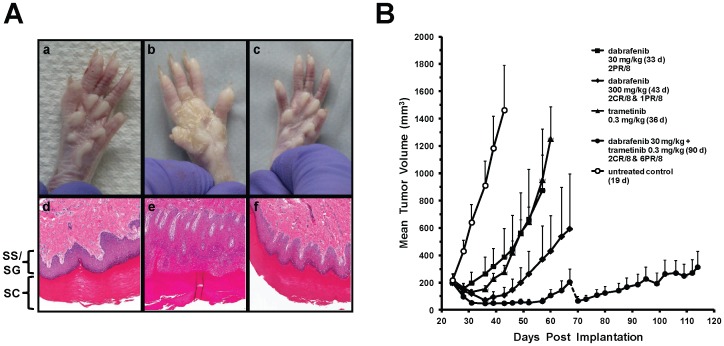
BRAF and MEK inhibitor combination decreases rat skin lesion formation and increases tumor growth inhibition. Macroscopic and light microscopic photographs of skin (ventral forepaw) from rats given vehicle (a/d), 150 mg/kg/d BRAF tool inhibitor (b/e), or 150 mg/kg/d BRAF tool inhibitor with 0.75 mg/kg/d MEK tool inhibitor (c/f) by oral gavage for 12 consecutive days (A). Stratum spinosum/stratum granulosum (epithelial layer, SS/SG) and stratum corneum (keratin layer, SC) are indicated. Mice bearing A375P tumors were treated orally, once daily, with the indicated doses of dabrafenib, trametinib, or a combination of both agents (B). Treatment continued until the mean tumor volume of each group reached 1 200 mm^3^ or one death occurred. Mean tumor volumes are plotted with their standard error mean and complete (CR) or partial (PR) regressions are indicated for each group of n = 8 mice after 14 days of treatment. The dotted line for the combination group indicates where one animal was euthanized due to tumor necrosis.

To test if concomitant treatment with our BRAF and MEK inhibitors would ameliorate BRAF inhibitor-induced skin hyperplasia, as demonstrated previously with PLX4720 and PD184352 [30], we performed a rat 12-day repeat dose investigative study with BRAF (GSK2366297) and MEK (GSK2091976 [31]) tool inhibitors. GSK2366297 is of similar structure and activity profile to dabrafenib and was selected due to a faster onset of skin lesions, likely related to its higher potency (Table S3). Macroscopic and light microscopic photographs (Figure 7A, panels a-c and d-f, respectively) of ventral forepaw skin were taken following treatment. Rats treated with GSK2366297 alone showed a generalized skin crusting with epithelial hyperplasia and hyperkeratosis (Figure 7A, b). Microscopically, these areas correlated with minimal to moderately increased epidermal thickening (hyperplasia), primarily affecting the stratum spinosum and to a lesser extent the stratum granulosum, with epithelial invagination into the dermis (rete ridge formation), and increased thickening of the overlaying keratinized stratum corneum (Figure 7A, e). The accompanying hyperkeratosis, primarily orthokeratotic, was characterized by increased stratum corneum thickening. Epithelial hyperplasia/hyperkeratosis was not observed in the control rats (Figure 7A, a/d), those given GSK2091976 alone (data not shown), or the combination of GSK2366297 with GSK2091976 (Figure 7A, c/f). Similar results were also seen in scrotal skin and forestomach, and pharmacokinetic data confirmed similar systemic exposure to GSK2366297 when delivered alone or in combination with GSK2091976 (data not shown). Phospho-ERK immunoreactivity within the epidermis was inconclusive due to staining variability, but Ki67 within the basal epithelial layer (paws and forestomach) was increased in rats given GSK2366297 alone, consistent with the observed epithelial hyperplasia (data not shown).

Development of these hyperproliferative lesions is likely pharmacologically mediated by paradoxical RAF inhibitor-dependent MAPK upregulation in wild-type RAF cells, which can enhance growth via ARAF or CRAF [20], [32]. This mechanism may support the development of SCC in humans treated with RAF inhibitors [33]. From data in Figure 6 we hypothesized that BRAF/MEK inhibitor co-administration would prevent MAPK upregulation and skin lesion development in rats. Indeed, *in vivo* data in Figure 7A confirmed that BRAF inhibitor-induced skin lesions in rats could be prevented by concomitant treatment with a MEK inhibitor. Together with our *in vitro* cellular data, these results are consistent with pharmacologically-mediated mechanisms suggestive of paradoxical MAPK pathway activation by dabrafenib in wild-type BRAF cells.

Finally, we investigated whether BRAF and MEK inhibitor co-administration would also be advantageous in reducing BRAF^V600E^ tumor growth. Figure 7B demonstrates that combined oral treatment for 90 consecutive days with dabrafenib and trametinib shows superior inhibition of tumor growth over either drug alone, including a 10-fold higher, single agent dose (300 mg/kg) providing the maximum bioavailable blood level of dabrafenib. At termination (day 43) of the vehicle-treated group, the beneficial effect of the combination on inhibition of tumor growth was significantly superior to 30 mg/kg dabrafenib alone (p = 0.01) and 0.3 mg/kg trametinib alone (p = 0.0001). Circulating blood levels for single and combined agents at day 1 and 58 showed similar concentrations, suggesting steady state.

## Discussion

BRAF-activating mutations have been identified in many cancers [6] as a causative determinant of hyperproliferation [8], [19]. RNA knockout [34] and compound inhibition of BRAF [35]–[37] causes cell cycle arrest and death of mutant BRAF tumor cells, mitigating the clinical use of BRAF inhibitors for treatment of activated BRAF-driven cancers.

We have characterized dabrafenib as a selective RAF kinase inhibitor with activity against full-length BRAF from multiple species. While dabrafenib inhibits a truncated CRAF kinase *in vitro*, this does not translate into cell culture, as cell lines with CRAF-dependent MEK activation (MEK inhibitor-sensitive) are insensitive to dabrafenib. The reason for this discrepancy between enzymatic and cellular data is unknown, but could be attributed to higher inhibitor sensitivity of truncated (enzyme assay) versus full-length (cellular) CRAF. Alternatively, cellular factors may exist that modify CRAF conformation, preventing its binding to dabrafenib. We also cannot exclude the possibility of a cellular compensatory mechanism, or a combination of all factors described above. We demonstrated high selectivity of dabrafenib for 80% of the BRAF^V600E^ cell lines tested and hypothesize that the relative lack of activity (gIC_50_>2 µM) against 3 of the BRAF^V600E^ cell lines may be due to the presence of additional mutations (PTEN in GCT cells, PI3K in RKO cells, and p53 in A673 cells) that were confirmed by in-house sequencing. Full sequencing data for these and other cell lines used in this study can be found at the Cancer Genome Workbench (CGWB) portal of the NCI (https://cgwb.nci.nih.gov/). Overexpression of proteins capable of driving cell growth/survival, or upregulation of efflux pump(s) could also serve to rescue cell growth from dabrafenib inhibition. While dabrafenib-sensitive BRAF^V600E^ cell lines also occasionally encode other mutations, we maintain that BRAF^V600E^ is the key oncogenic driver in these cells. We demonstrated that dabrafenib is also active against the activated BRAF^V600K^ and BRAF^V600D^ mutants at both the enzyme and cellular level. This is supported by recent clinical observations where patients with BRAF^V600D/E/K^ tumors responded to GSK2118436 (dabrafenib) treatment [38]. We also observed slight dabrafenib sensitivity (gIC_50_ from 263 nM to 6.9 µM) of 18 cell lines lacking a BRAF-activating mutation and containing wild-type RAS. While no common mutations were identified between these cell lines, we speculate that activating mutation or overexpression of upstream BRAF drivers could result in BRAF-dependent cell proliferation. Additionally, while dabrafenib is very selective, we can’t exclude off-target effects that may result in sensitivity to dabrafenib.

Dabrafenib shows specific activity for cells containing activated mutant BRAF. In BRAF^V600E^ cells, dabrafenib inhibits MEK and ERK activation in a concentration-dependent manner, with similar, low nM activity (Figure S2). We confirmed that in a BRAF^V600E^ cell line (A375P), ARAF or CRAF knockdown had no effect on MAPK activation. However, dabrafenib inhibited pMEK and pERK in a manner similar to that caused by BRAF depletion, confirming an on-target mechanism for dabrafenib as MAPK activation is BRAF-dependent in BRAF^V600E^ cells. Surprisingly, ARAF or CRAF depletion diminished MAPK inhibition by dabrafenib, suggesting that ARAF or CRAF absence may allow increased formation of highly active BRAF^V600E^ homodimers, and fewer ARAF/BRAF^V600E^ or CRAF/BRAF^V600E^ heterodimers. Alternatively, ARAF or CRAF depletion might induce a relaxation of negative feedback loops. Slightly lower levels of MEK and ERK were observed with BRAF siRNA in lanes 4 and 7, but not with dabrafenib alone in lane 2, despite its ability to induce cell death. However, cells were only exposed to dabrafenib for 1 h, which is not sufficient to reduce intact protein content through apoptosis, while siRNA treatment was for 72 h. In summary, these data clearly support the specific cellular selectivity of dabrafenib for activated mutant BRAF in cancer cells.

*In vivo* pharmacodynamic marker modulation and tumor growth inhibition by dabrafenib were demonstrated in human BRAF^V600E^ tumor xenograft models. ERK inhibition was rapid (2 h post-dosing) and surprisingly sustained at times (18 h post-dosing on days 7 and 14) when the circulating dabrafenib concentration was below that required for inhibition in cell culture. This could not be explained by tumor accumulation of the drug (data not shown), but might be attributed to the presence of circulating active dabrafenib metabolites. *In vivo* inhibition of MAPK signalling correlated with decreased tumor cell growth and dose-dependent tumor regression was observed with continuous drug exposure, supporting an on-target mechanism for dabrafenib.

We demonstrated that dabrafenib could elevate pMEK and pERK in a CRAF-dependent manner in cells encoding mutant RAS, but wild-type RAF, and which are also dependent on the MAPK pathway for growth (sensitive to MEK inhibition). Paradoxical activation of MAPK signalling by BRAF inhibitors has been shown to be dependent upon RAS activity [22], where only one member of the RAF dimer is inhibited. It is therefore likely that dabrafenib-inhibited wild-type BRAF or CRAF in HCT-116 cells is binding to an uninhibited wild-type CRAF protomer in a RAS-dependent manner, resulting in elevated MAPK signalling. Since our data in HCT-116 cells (Figure 6) showed no effect with BRAF siRNA but abolition of dabrafenib-induced MAPK signalling by CRAF siRNA, this further supports the notion of CRAF dependence for paradoxical MAPK activation upon BRAF inhibitor treatment in an activated (mutant) RAS cell type. Since dabrafenib could elevate MAPK signalling in wild-type BRAF cells, we hypothesized that this could cause uncontrolled skin cell growth, leading to abnormalities such as the SCC as observed in patients treated with BRAF inhibitors [39]. We demonstrated BRAF inhibitor-induced occurrence of skin lesions in a rat model using a structurally similar tool compound to dabrafenib and showed that co-treatment with a MEK inhibitor could prevent occurrence of this pathology. In addition to a reduced potential for BRAF inhibitor-activated growth of wild-type RAF cells, the combination of dabrafenib with a MEK inhibitor (trametinib) showed enhanced efficacy in a BRAF^V600E^ tumor xenograft model. Although dabrafenib administration was sustained (33 days at 30 mg/kg and 43 days at 300 mg/kg), tumor growth inhibition was observed only for a short period (14 and 20 days, respectively), followed by tumor re-growth, albeit at a slower rate than in untreated animals (Figure 7B), suggesting that a resistance mechanism develops upon constant drug exposure. Circulating drug in animals treated with 300 mg/kg dabrafenib was similar in concentration and profile at days 1, 70, and 84 (data not shown), suggesting that tumor re-growth did not result from time-dependent activation of dabrafenib metabolism. In a separate study (not shown), tumors growing upon extended dabrafenib treatment subsequently responded to a MEK inhibitor (trametinib), suggesting that the *in vivo* dabrafenib resistance mechanism in this experiment was ERK-dependent. Similar resistance mechanisms have been observed by others [40]–[43], including the recently identified involvement of a BRAF^V600E^ p61 splice variant [44]. The possibility of an off-target mechanism, such as primary immune dysregulation, causing the observed BRAF inhibitor-induced skin hyperplasia also must not be ignored. However, the role of paradoxically activated MAPK signalling seems very likely given similar results with other RAF inhibitors of different chemical series [14]–[18]. Furthermore, recent studies have also shown an association between RAS mutation and SCC/keratoacanthoma formation in patients treated with RAF inhibitors [30], [45]. Consequently, we hypothesize that in clinical trials the MEK inhibitor concentration needed to inhibit undesired BRAF inhibitor-induced wild-type BRAF cell growth may also be lower than that required for single agent activity, reducing the potential for undesirable toxicity from either inhibitor, including a reduction in SCC incidence. To this end, clinical trials to test the combination of both agents dabrafenib (GSK2118436) and trametinib (GSK1120212) are currently ongoing in patients with melanoma. Recent data from a phase 1/2 trial have indeed shown that the combination of dabrafenib with trametinib at full monotherapy doses significantly improved progression-free survival (p<0.001) and the rate of complete or partial responses observed (p = 0.03) [46]. Furthermore, the occurrence of skin lesions was also reduced, as hypothesized, albeit nonsignificantly (p = 0.09) and with increased pyrexia. However, taken together this combination provides tremendous promise for patients with tumors expressing oncogenic mutant BRAF.

## Supporting Information

Figure S1 **Dabrafenib binding to BRAF^V600E^ and wild-type BRAF is ATP-competitive.** ATP-competitive FP ligand (1 nM) was mixed with various concentrations of BRAF^V600E^ (A) or wild-type (WT) BRAF (B) for 60 min and fluorescence polarization (mP) values were fitted to determine ligand K_d_ values. BRAF^V600E^ (C) or WT BRAF (D) were mixed with FP ligand and various concentrations of dabrafenib, incubated for 60 min to reach equilibrium, and fluorescence polarization (mP) values were measured to show competition with FP ligand binding. IC_50_ values were determined as 0.68 nM and 0.64 nM for BRAF^V600E^ and WT BRAF, respectively.
(PPTX)

Figure S2 **Dabrafenib inhibits pERK and pMEK in a concentration-dependent manner.** ES-2 ovarian carcinoma (BRAF^V600E^) cells were treated for 1 h with dabrafenib and immunoblotted for phospho-ERK1/2 (pT202/pY204; pERK), total ERK1/2 (tERK), phospho-MEK1/2 (pMEK), and total MEK1/2 (tMEK). Signals were quantified and used to determine IC_50_ values.
(PPTX)

Figure S3 **Dabrafenib inhibits BRAF^V600E^ cell proliferation through a G_1_ arrest and causes caspase-3/7 activation.** A375P and SK-MEL-28 melanoma (BRAF^V600E^) cells and Human Foreskin Fibroblasts (HFF, wild-type BRAF) were analyzed for cell cycle profile by DNA content using flow cytometry (A) or caspase-3/7 activation using Caspase-Glo® reagent (B), following a 72-hour exposure to dabrafenib or DMSO control. Cell cycle phases are shown in stacked format as a percentage of the total population. The dabrafenib concentration required to induce a 2-fold (200%) capase-3/7 activation over DMSO control (EC_200_) is shown for each cell line.
(PPTX)

Table S1 **Dabrafenib selectively inhibits BRAF and CRAF kinases.** Dabrafenib was tested against 270 kinases (Millipore) at 3 µM and 300 nM. Enzyme activity IC_50_ values were determined for kinases with >60% inhibition at 300 nM dabrafenib and those with IC_50_ values <100 nM are shown above. *A binding assay was used to measure ALK5 activity. Cell-based assay data showed an absence of ALK5 inhibition by dabrafenib.
(PDF)

Table S2 **Inhibition of tumor cell growth by dabrafenib.** Cell growth inhibition by dabrafenib was tested between 0.02 nM and 10 µM against 195 cell lines in a 3-day assay using CellTiter-Glo® readout. The dabrafenib concentration causing 50% growth inhibition (gIC_50_) is reported for each cell line, along with RAF and RAS gene mutational status.
(PDF)

Table S3 **Enzymatic and cellular activity of tool inhibitors used in the rat skin lesion formation study.** Activity of GSK2366297A (BRAF tool inhibitor) and GSK2091975 (MEK tool inhibitor, active form of prodrug GSK2091976) were measured against their respective enzyme targets as described in the Materials and Methods section. Cellular activity was determined for each by inhibition of ERK phosphorylation in SK-MEL-28 cells and growth of A375 cells, both of which express BRAF^V600E^.
(PDF)

## References

[1] Yoon S, Seger R (2006) The extracellular signal-regulated kinase: multiple substrates regulate diverse cellular functions. Growth Factors 24: 21–44. 16393692 10.1080/02699050500284218

[2] Dhillon AS, Hagan S, Rath O, Kolch W (2007) MAP kinase signalling pathways in cancer. Oncogene 26: 3279–3290. 17496922 10.1038/sj.onc.1210421

[3] Montagut C, Settleman J (2009) Targeting the RAF-MEK-ERK pathway in cancer therapy. Cancer Lett 283: 125–134. 19217204 10.1016/j.canlet.2009.01.022

[4] Young A, Lyons J, Miller AL, Phan VT, Alarcon IR, et al (2009) Ras signaling and therapies. Adv Cancer Res 102: 1–17. 19595305 10.1016/S0065-230X(09)02001-6

[5] Eggermont AM, Robert C (2011) New drugs in melanoma: it's a whole new world. Eur J Cancer 47: 2150–2157. 21802280 10.1016/j.ejca.2011.06.052

[6] Davies H, Bignell GR, Cox C, Stephens P, Edkins S, et al (2002) Mutations of the BRAF gene in human cancer. Nature 417: 949–954. 12068308 10.1038/nature00766

[7] Wellbrock C, Karasarides M, Marais R (2004) The RAF proteins take centre stage. Nat Rev Mol Cell Biol 5: 875–885. 15520807 10.1038/nrm1498

[8] Wan PT, Garnett MJ, Roe SM, Lee S, Niculescu-Duvaz D, et al (2004) Mechanism of activation of the RAF-ERK signaling pathway by oncogenic mutations of B-RAF. Cell 116: 855–867. 15035987 10.1016/s0092-8674(04)00215-6

[9] Wilhelm SM, Adnane L, Newell P, Villanueva A, Llovet JM, et al (2008) Preclinical overview of sorafenib, a multikinase inhibitor that targets both Raf and VEGF and PDGF receptor tyrosine kinase signaling. Mol Cancer Ther 7: 3129–3140. 18852116 10.1158/1535-7163.MCT-08-0013 PMC12261297

[10] Bollag G, Hirth P, Tsai J, Zhang J, Ibrahim PN, et al (2010) Clinical efficacy of a RAF inhibitor needs broad target blockade in BRAF-mutant melanoma. Nature 467: 596–599. 20823850 10.1038/nature09454 PMC2948082

[11] Kefford R, Arkenau H, Brown MP, Millward M, Infante JR, et al.. (2010) Phase I/II study of GSK2118436, a selective inhibitor of oncogenic mutant BRAF kinase, in patients with metastatic melanoma and other solid tumors. J Clin Oncol 28(15 suppl): abstr 8503.

[12] Long GV, Menzies AM, Nagrial AM, Haydu LE, Hamilton AL, et al (2011) Prognostic and clinicopathologic associations of oncogenic BRAF in metastatic melanoma. J Clin Oncol 29: 1239–1246. 21343559 10.1200/JCO.2010.32.4327

[13] Sharfman WH, Hodi FS, Lawrence DP, Flaherty KT, Amaravadi RK, et al.. (2011) Results from the first-in-human (FIH) phase I study of the oral RAF inhibitor RAF265 administered daily to patients with advanced cutaneous melanoma. J Clin Oncol 29(15 suppl): abstr 8508.

[14] Hong DS, Reddy SB, Prieto VG, Wright JJ, Tannir NM, et al (2008) Multiple squamous cell carcinomas of the skin after therapy with sorafenib combined with tipifarnib. Arch Dermatol 144: 779–782. 18559769 10.1001/archderm.144.6.779

[15] Kong HH, Sibaud V, Chanco Turner ML, Foto J, Hornyak TJ, et al (2008) Sorafenib-induced eruptive melanocytic lesions. Arch Dermatol 144: 820–822. 18559790 10.1001/archderm.144.6.820 PMC2556207

[16] Dubauskas Z, Kunishige J, Prieto VG, Jonasch E, Hwu P, et al (2009) Cutaneous squamous cell carcinoma and inflammation of actinic keratoses associated with sorafenib. Clin Genitourin Cancer 7: 20–23. 19213663 10.3816/CGC.2009.n.003 PMC4825856

[17] Arnault JP, Wechsler J, Escudier B, Spatz A, Tomasic G, et al (2009) Keratoacanthomas and squamous cell carcinomas in patients receiving sorafenib. J Clin Oncol 27: e59–e61. 19597016 10.1200/JCO.2009.23.4823

[18] Flaherty K, Puzanov I, Sosman J, Kim K, Ribas A, et al.. (2009) Phase I study of PLX4032: proof of concept for V600E BRAF mutation as a therapeutic target in human cancer. J Clin Oncol 27(15 suppl): abstr 9000.

[19] Cichowski K, Jänne PA (2010) Drug discovery: inhibitors that activate. Nature 464: 358–359. 20237552 10.1038/464358a

[20] Hatzivassiliou G, Song K, Yen I, Brandhuber BJ, Anderson DJ, et al (2010) RAF inhibitors prime wild-type RAF to activate the MAPK pathway and enhance growth. Nature 464: 431–435. 20130576 10.1038/nature08833

[21] Heidorn SJ, Milagre C, Whittaker S, Nourry A, Niculescu-Duvas I, et al (2010) Kinase-dead BRAF and oncogenic RAS cooperate to drive tumor progression through CRAF. Cell 140: 209–221. 20141835 10.1016/j.cell.2009.12.040 PMC2872605

[22] Poulikakos PI, Zhang C, Bollag G, Shokat KM, Rosen N (2010) RAF inhibitors transactivate RAF dimers and ERK signalling in cells with wild-type BRAF. Nature 464: 427–430. 20179705 10.1038/nature08902 PMC3178447

[23] Rheault TR, Stellwagen JC, Adjabeng GM, Hornberger KR, Petrov KG, et al (2013) Discovery of dabrafenib: A selective inhibitor of Raf kinases with anti-tumor activity against B-Raf-driven tumors. ACS Med Chem Lett 4: 358–362. 24900673 10.1021/ml4000063 PMC4027516

[24] Rominger CM, Schaber MD, Yang J, Gontarek RR, Weaver KL, et al (2007) An intrinsic ATPase activity of phospho-MEK-1 uncoupled from downstream ERK phosphorylation. Arch Biochem Biophys 464: 130–137. 17490600 10.1016/j.abb.2007.04.004

[25] Vindeløv LL (1977) Flow microfluorometric analysis of nuclear DNA in cells from solid tumors and cell suspensions. A new method for rapid isolation and staining of nuclei. Virchows Arch B Cell Pathol 24: 227–242. 410154

[26] Hingorani SR, Jacobetz MA, Robertson GP, Herlyn M, Tuveson DA (2003) Suppression of BRAF(V599E) in human melanoma abrogates transformation. Cancer Res 63: 5198–5202. 14500344

[27] Wellbrock C, Ogilvie L, Hedley D, Karasarides M, Martin J, et al (2004) ^V599E^B-RAF is an oncogene in melanocytes. Cancer Res 64: 2338–2342. 15059882 10.1158/0008-5472.can-03-3433

[28] Xing F, Persaud Y, Pratilas CA, Taylor BS, Janakiraman M, et al (2012) Concurrent loss of the PTEN and RB1 tumor suppressors attenuates RAF dependence in melanomas harboring (V600E)BRAF. Oncogene 31: 446–457. 21725359 10.1038/onc.2011.250 PMC3267014

[29] Gilmartin AG, Bleam MR, Groy A, Moss KG, Minthorn EA, et al (2011) GSK1120212 (JTP-74057) is an inhibitor of MEK activity and activation with favorable pharmacokinetic properties for sustained in vivo pathway inhibition. Clin Cancer Res 17: 989–1000. 21245089 10.1158/1078-0432.CCR-10-2200

[30] Su F, Viros A, Milagre C, Trunzer K, Bollag G, et al (2012) RAS mutations in cutaneous squamous-cell carcinomas in patients treated with BRAF inhibitors. New Engl J Med 366: 207–215. 22256804 10.1056/NEJMoa1105358 PMC3724537

[31] Ralph JM, Adams JL, Silva DJ, Feng Y, Martin PJ, et al. (2011) Evaluation of N-acyl sulfonamide prodrug inhibitors of MEK kinase. American Chemical Society; Division of Medicinal Chemistry, 242^nd^ ACS National Meeting: abstr. MEDI 34.

[32] Carnahan J, Beltran PJ, Babij C, Le Q, Rose MJ, et al (2010) Selective and potent Raf inhibitors paradoxically stimulate normal cell proliferation and tumor growth. Mol Cancer Ther 9: 2399–2410. 20663930 10.1158/1535-7163.MCT-10-0181

[33] Garnett MJ, Marais R (2004) Guilty as charged: B-RAF is a human oncogene. Cancer Cell 6: 313–319. 15488754 10.1016/j.ccr.2004.09.022

[34] Karasarides M, Chiloeches A, Hayward R, Niculescu-Duvaz D, Scanlon I, et al (2004) B-RAF is a therapeutic target in melanoma. Oncogene 23: 6292–6298. 15208680 10.1038/sj.onc.1207785

[35] Sharma A, Trivedi NR, Zimmerman MA, Tuveson DA, Smith CD, et al (2005) Mutant V599EB-Raf regulates growth and vascular development of malignant melanoma tumors. Cancer Res 65: 2412–2421. 15781657 10.1158/0008-5472.CAN-04-2423

[36] King AJ, Patrick DR, Batorsky RS, Ho ML, Do HT, et al (2006) Demonstration of a genetic therapeutic index for tumors expressing oncogenic BRAF by the kinase inhibitor SB-590885. Cancer Res 66: 11100–11105. 17145850 10.1158/0008-5472.CAN-06-2554

[37] Tsai J, Lee JT, Wang W, Zhang J, Cho H, et al (2008) Discovery of a selective inhibitor of oncogenic B-Raf kinase with potent antimelanoma activity. Proc Natl Acad Sci USA 105: 3041–3046. 18287029 10.1073/pnas.0711741105 PMC2268581

[38] Kefford R, Long G, Arkenau HT, Brown MP, Millward M, et al (2010) Selective inhibition of oncogenic BRAF V600E/K/D by GSK2118436: evidence of clinical activity in subjects with metastatic melanoma. Pigment Cell Melanoma Res 23: 874–1004.

[39] Chapman PB, Hauschild A, Robert C, Haanen JB, Ascierto P, et al (2011) Improved survival with vemurafenib in melanoma with BRAF V600E mutation. N Engl J Med 364: 2507–2516. 21639808 10.1056/NEJMoa1103782 PMC3549296

[40] Johannessen CM, Boehm JS, Kim SY, Thomas SR, Wardwell L, et al (2010) COT drives resistance to RAF inhibition through MAP kinase pathway reactivation. Nature 468: 968–972. 21107320 10.1038/nature09627 PMC3058384

[41] Nazarian R, Shi H, Wang Q, Kong X, Koya RC, et al (2010) Melanomas acquire resistance to B-RAF(V600E) inhibition by RTK or N-RAS upregulation. Nature 468: 973–977. 21107323 10.1038/nature09626 PMC3143360

[42] Corcoran RB, Settleman J, Engelman JA (2011) Potential therapeutic strategies to overcome acquired resistance to BRAF or MEK inhibitors in BRAF mutant cancers. Oncotarget 2: 336–346. 21505228 10.18632/oncotarget.262 PMC3248170

[43] Wagle N, Emery C, Berger MF, Davis MJ, Sawyer A, et al (2011) Dissecting therapeutic resistance to RAF inhibition in melanoma by tumor genomic profiling. J Clin Oncol 29: 3085–3096. 21383288 10.1200/JCO.2010.33.2312 PMC3157968

[44] Poulikakos PI, Persaud Y, Janakiraman M, Kong X, Ng C, et al (2011) RAF inhibitor resistance is mediated by dimerization of aberrantly spliced BRAF (V600E). Nature 480: 387–390. 22113612 10.1038/nature10662 PMC3266695

[45] Oberholzer PA, Kee D, Dziunycz P, Sucker A, Kamsukom N, et al (2012) RAS mutations are associated with the development of cutaneous squamous cell tumors in patients treated with RAF inhibitors. J Clin Oncol 30: 316–321. 22067401 10.1200/JCO.2011.36.7680 PMC3269955

[46] Flaherty KT, Infante JR, Daud A, Gonzalez R, Kefford RF, et al (2012) Combined BRAF and MEK inhibition in melanoma with BRAF V600 mutations. N Engl J Med 367: 1694–1703. 23020132 10.1056/NEJMoa1210093 PMC3549295

