# Supplementary figures and images for: Dabrafenib; Preclinical Characterization, Increased Efficacy when Combined with Trametinib, while BRAF/MEK Tool Combination Reduced Skin Lesions
Source: PLoS One. 2013 Jul 3;8(7):e67583. doi: 10.1371/journal.pone.0067583 (PMC3701070; doi:10.1371/journal.pone.0067583)

## Slide 1
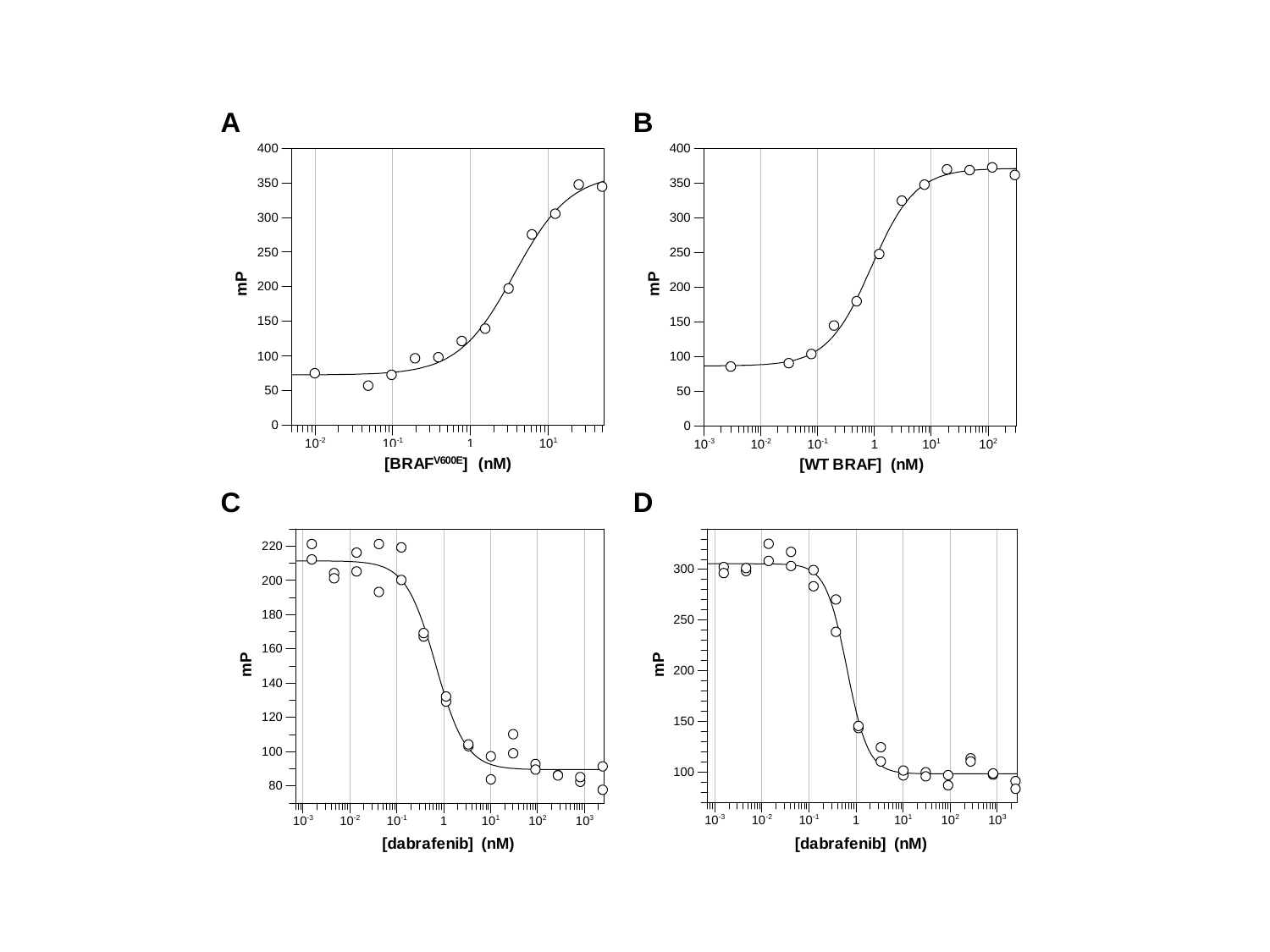

Supplement: Figure S1 — Dabrafenib binding to BRAFV600E and wild-type BRAF is ATP-competitive. ATP-competitive FP ligand (1 nM) was mixed with various concentrations of BRAFV600E (A) or wild-type (WT) BRAF (B) for 60 min and fluorescence polarization (mP) values were fitted to determine ligand Kd values. BRAFV600E (C) or WT BRAF (D) were mixed with FP ligand and various concentrations of dabrafenib, incubated for 60 min to reach equilibrium, and fluorescence polarization (mP) values were measured to show competition with FP ligand binding. IC50 values were determined as 0.68 nM and 0.64 nM for BRAFV600E and WT BRAF, respectively. (PPTX) [file pone.0067583.s001.pptx]

## Slide 1
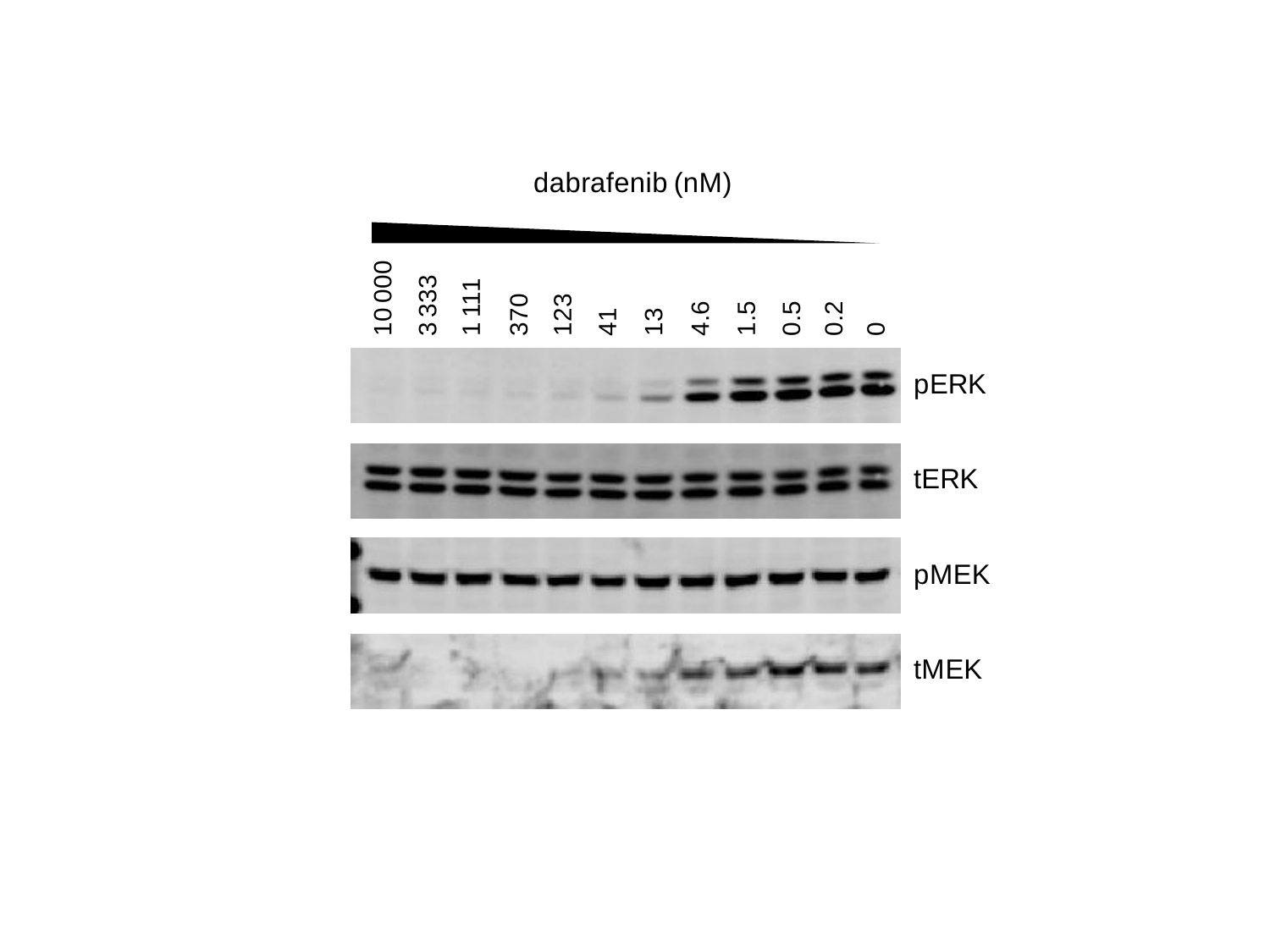

Supplement: Figure S2 — Dabrafenib inhibits pERK and pMEK in a concentration-dependent manner. ES-2 ovarian carcinoma (BRAFV600E) cells were treated for 1 h with dabrafenib and immunoblotted for phospho-ERK1/2 (pT202/pY204; pERK), total ERK1/2 (tERK), phospho-MEK1/2 (pMEK), and total MEK1/2 (tMEK). Signals were quantified and used to determine IC50 values. (PPTX) [file pone.0067583.s002.pptx]

## Slide 1
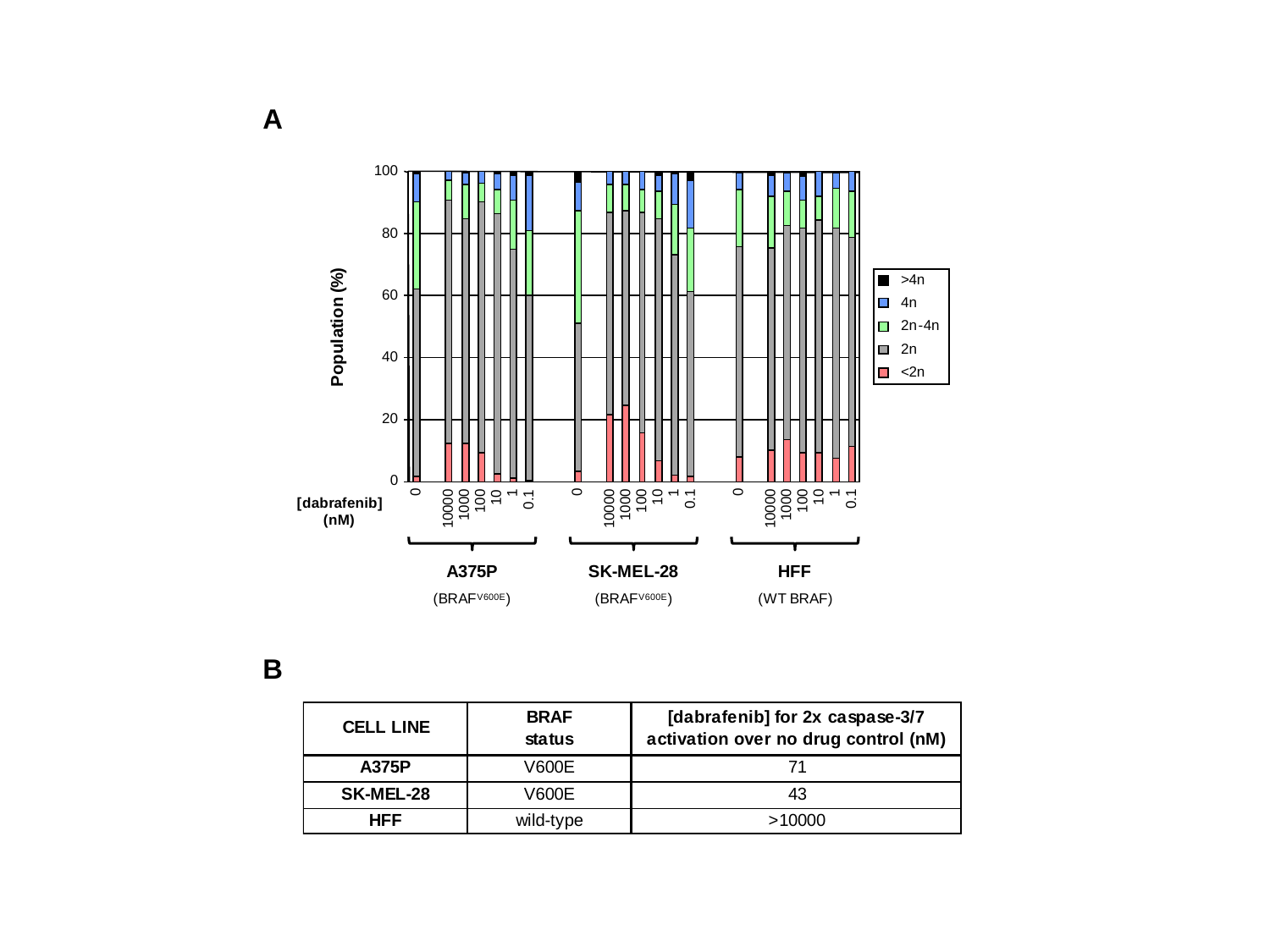

Supplement: Figure S3 — Dabrafenib inhibits BRAFV600E cell proliferation through a G1 arrest and causes caspase-3/7 activation. A375P and SK-MEL-28 melanoma (BRAFV600E) cells and Human Foreskin Fibroblasts (HFF, wild-type BRAF) were analyzed for cell cycle profile by DNA content using flow cytometry (A) or caspase-3/7 activation using Caspase-Glo® reagent (B), following a 72-hour exposure to dabrafenib or DMSO control. Cell cycle phases are shown in stacked format as a percentage of the total population. The dabrafenib concentration required to induce a 2-fold (200%) capase-3/7 activation over DMSO control (EC200) is shown for each cell line. (PPTX) [file pone.0067583.s003.pptx]
