# Supplementary material for: Dabrafenib; Preclinical Characterization, Increased Efficacy when Combined with Trametinib, while BRAF/MEK Tool Combination Reduced Skin Lesions
Source: PLoS One. 2013 Jul 3;8(7):e67583. doi: 10.1371/journal.pone.0067583 (PMC3701070; doi:10.1371/journal.pone.0067583)
Supplement: Table S1 — Dabrafenib selectively inhibits BRAF and CRAF kinases. Dabrafenib was tested against 270 kinases (Millipore) at 3 µM and 300 nM. Enzyme activity IC50 values were determined for kinases with >60% inhibition at 300 nM dabrafenib and those with IC50 values <100 nM are shown above. *A binding assay was used to measure ALK5 activity. Cell-based assay data showed an absence of ALK5 inhibition by dabrafenib. (PDF) [file pone.0067583.s004.pdf]

**KINASE**	**IC_{50} (nM)**
BRAF	3.2
CRAF	5.0
BRK (h)	79
CK1 (y)	41
LIMK1 (h)	15
NEK11 (h)	20
PKD2 (h)	57
SIK (h)	27
ALK5 (h)*	11
