# Supplementary material for: Dabrafenib; Preclinical Characterization, Increased Efficacy when Combined with Trametinib, while BRAF/MEK Tool Combination Reduced Skin Lesions
Source: PLoS One. 2013 Jul 3;8(7):e67583. doi: 10.1371/journal.pone.0067583 (PMC3701070; doi:10.1371/journal.pone.0067583)
Supplement: Table S2 — Inhibition of tumor cell growth by dabrafenib. Cell growth inhibition by dabrafenib was tested between 0.02 nM and 10 µM against 195 cell lines in a 3-day assay using CellTiter-Glo® readout. The dabrafenib concentration causing 50% growth inhibition (gIC50) is reported for each cell line, along with RAF and RAS gene mutational status. (PDF) [file pone.0067583.s005.pdf]

CELL LINE	RAF	RAS	gIC$_{50}$ (nM)	CELL LINE	RAF	RAS	gIC$_{50}$ (nM)	CELL LINE	RAF	RAS	gIC$_{50}$ (nM)
MALME-3M	BRAF(V600E)	WT	1	647-V	WT	WT	10000	NCI-H226	WT	WT	10000
UACC-62	BRAF(V600E)	WT	1	786-O	WT	WT	10000	NCI-H292	WT	WT	10000
C32TG	BRAF(V600E)	WT	1	A172	WT	WT	10000	NCI-H322	WT	WT	10000
SK-MEL-1	BRAF(V600E)	WT	2	A204	WT	WT	10000	NCI-H358	WT	WT	10000
M14	BRAF(V600E)	WT	2	A427	WT	WT	10000	NCI-H520	WT	WT	10000
SK-MEL-28	BRAF(V600E)	WT	3	A431	WT	WT	10000	NCI-H526	WT	WT	10000
A375	BRAF(V600E)	WT	4	A2780	WT	WT	10000	NCI-H630	WT	WT	10000
DU-4475	BRAF(V600E)	WT	5	ARH-77	WT	WT	10000	NCI-H661	WT	WT	10000
UACC-257	BRAF(V600E)	WT	6	Be(2)C	WT	WT	10000	NCI-H716	WT	WT	10000
Colo 205	BRAF(V600E)	WT	7	BT-20	WT	WT	10000	NCI-H1563	WT	WT	10000
SK-MEL-3	BRAF(V600E)	WT	7	BxPc3	WT	WT	10000	NCI-H1792	WT	WT	10000
SH-4	BRAF(V600E)	WT	8	C-4 I	WT	WT	10000	NCI-H2030	WT	WT	10000
A101D	BRAF(V600E)	WT	9	C33A	WT	WT	10000	NCI-H2052	WT	WT	10000
ES-2	BRAF(V600E)	WT	53	CAL-27	WT	WT	10000	NCI-H2122	WT	WT	10000
HT-29	BRAF(V600E)	WT	66	CAL-62	WT	WT	10000	NCI-H2228	WT	WT	10000
SW1417	BRAF(V600E)	WT	158	Calu-3	WT	WT	10000	OE-19	WT	WT	10000
SW872	BRAF(V600E)	WT	377	CaOV3	WT	WT	10000	OE-21	WT	WT	10000
RKO	BRAF(V600E)	WT	2522	CGTHW1	WT	WT	10000	OE-33	WT	WT	10000
A673	BRAF(V600E)	WT	10000	CHL-1	WT	WT	10000	OVCAR-3	WT	WT	10000
GCT	BRAF(V600E)	WT	10000	Colo320DM	WT	WT	10000	OVCAR-4	WT	WT	10000
				Colo704	WT	WT	10000	OVCAR-5	WT	WT	10000
WM-115	BRAF(V600D)	WT	5	COR-L23	WT	WT	10000	P3HR-1	WT	WT	10000
YUMAC	BRAF(V600K)	WT	5	CRO-AP2	WT	WT	10000	PC-3	WT	WT	10000
OV-90	BRAF(del486-490)	WT	29	Daudi	WT	WT	10000	Raji	WT	WT	10000
NCI-H508	BRAF(G596R)	WT	10000	DOTC 24510	WT	WT	10000	RD	WT	WT	10000
MDA-MB-231	BRAF(G464V)	KRAS(G13D)	10000	DU-145	WT	WT	10000	RD-ES	WT	WT	10000
				EB-3	WT	WT	10000	RL	WT	WT	10000
SW756	WT	KRAS(G12C)	10000	EFM-19	WT	WT	10000	SaOS2	WT	WT	10000
SW-837	WT	KRAS(G12C)	10000	EKVX	WT	WT	10000	SCC-9	WT	WT	10000
UMUC-3	WT	KRAS(G12C)	10000	FaDu	WT	WT	10000	SCC-12	WT	WT	10000
LS-174T	WT	KRAS(G12D)	10000	G401	WT	WT	10000	SCC-13	WT	WT	10000
A549	WT	KRAS(G12S)	10000	HCC-70	WT	WT	10000	SCC-15	WT	WT	10000
SHP-77	WT	KRAS(G12V)	10000	HCC-1954	WT	WT	10000	SCC-25	WT	WT	10000
SW480	WT	KRAS(G12V)	10000	HeLa	WT	WT	10000	SCLC-3	WT	WT	10000
DLD-1	WT	KRAS(G13D)	10000	Hep3B	WT	WT	10000	SF-268	WT	WT	10000
HCT-116	WT	KRAS(G13D)	10000	HMCB	WT	WT	10000	SF-295	WT	WT	10000
NCI-H747	WT	KRAS(G13D)	10000	HN5	WT	WT	10000	SJRH30	WT	WT	10000
T-84	WT	KRAS(G13D)	10000	HOS	WT	WT	10000	SK-BR-3	WT	WT	10000
NCI-H460	WT	KRAS(Q61H)	10000	HPAF-II	WT	WT	10000	SK-LMS-1	WT	WT	10000
Calu-6	WT	KRAS(Q61K)	10000	Hs746.T	WT	WT	10000	SK-N-DZ(note)	WT	WT	10000
HCC-2998	WT	KRAS(A146T)	10000	HT	WT	WT	10000	SK-N-F1	WT	WT	10000
LS1034	WT	KRAS(A146T)	10000	HT-3	WT	WT	10000	SKOV-3	WT	WT	10000
CHP-212	WT	NRAS(Q61K)	10000	HuNS-1	WT	WT	10000	SK-UT-1	WT	WT	10000
SK-N-AS	WT	NRAS(Q61K)	10000	IGROV1	WT	WT	10000	SN12C	WT	WT	10000
BFTC-905	WT	NRAS(Q61L)	10000	J82	WT	WT	10000	SNB-19	WT	WT	10000
				JM1	WT	WT	10000	SNU-5	WT	WT	10000
HuT78	WT	WT	52	Kato III	WT	WT	10000	SNU-16	WT	WT	10000
RPMI-8226	WT	WT	263	KHOS-240S	WT	WT	10000	SNU-398	WT	WT	10000
BC-3	WT	WT	277	KM-12	WT	WT	10000	SNU-449	WT	WT	10000
ACHN	WT	WT	294	KPL-1	WT	WT	10000	SR	WT	WT	10000
JRT3-T35	WT	WT	295	KYSE-30	WT	WT	10000	ST486	WT	WT	10000
SK-MES-1	WT	WT	329	LNCaP	WT	WT	10000	SW579	WT	WT	10000
CESS	WT	WT	497	MCF-7	WT	WT	10000	SW684	WT	WT	10000
H4	WT	WT	619	MC-IXC	WT	WT	10000	SW-780	WT	WT	10000
BC-1	WT	WT	704	MDA-MB-175 VII	WT	WT	10000	SW900	WT	WT	10000
DB	WT	WT	873	MDA-MB-468	WT	WT	10000	SW1088	WT	WT	10000
RPMI-6666	WT	WT	928	MiaPaCa	WT	WT	10000	SW1463	WT	WT	10000
MC/CAR	WT	WT	1002	Molt-4	WT	WT	10000	SW1990	WT	WT	10000
SNU-1	WT	WT	1501	NCI-ADR/RES	WT	WT	10000	T47D	WT	WT	10000
CEM/C1	WT	WT	1773	NCI-H69	WT	WT	10000	TE381.T	WT	WT	10000
GDM-1	WT	WT	1997	NCI-H82	WT	WT	10000	TK-10	WT	WT	10000
HT-1080	WT	WT	2004	NCI-N87	WT	WT	10000	U2OS	WT	WT	10000
HL-60	WT	WT	2178	NCI-H146	WT	WT	10000	U251	WT	WT	10000
C3A	WT	WT	5414	NCI-H157	WT	WT	10000	UACC-812	WT	WT	10000
MES-SA	WT	WT	6989	NCI-H187	WT	WT	10000	YAPC	WT	WT	10000
22Rv1	WT	WT	10000	NCI-H209	WT	WT	10000	ZR-75-1	WT	WT	10000
