# Supplementary material for: Dabrafenib; Preclinical Characterization, Increased Efficacy when Combined with Trametinib, while BRAF/MEK Tool Combination Reduced Skin Lesions
Source: PLoS One. 2013 Jul 3;8(7):e67583. doi: 10.1371/journal.pone.0067583 (PMC3701070; doi:10.1371/journal.pone.0067583)
Supplement: Table S3 — Enzymatic and cellular activity of tool inhibitors used in the rat skin lesion formation study. Activity of GSK2366297A (BRAF tool inhibitor) and GSK2091975 (MEK tool inhibitor, active form of prodrug GSK2091976) were measured against their respective enzyme targets as described in the Materials and Methods section. Cellular activity was determined for each by inhibition of ERK phosphorylation in SK-MEL-28 cells and growth of A375 cells, both of which express BRAFV600E. (PDF) [file pone.0067583.s006.pdf]

IC_{50} (nM)	BRAF Tool Inhibitor<br>GSK2366297A	MEK Tool Inhibitor<br>GSK2091975 (activated drug)
**BRAF Enzyme**	0.20	>10000
**U-MEK Enzyme**	>10000	4.4
**pERK (SK-MEL28)**	2.0	11
**A375 Cell Growth (gIC_{50})**	9.5	2.1
